# QTc and QTd in Children with Type 1 Diabetes Mellitus during Diabetic Ketoacidosis

**DOI:** 10.5402/2012/619107

**Published:** 2012-11-06

**Authors:** Omneya I. Youssef, Samar M. Farid

**Affiliations:** Pediatric Department, Faculty of Medicine, Ain Shams University, Cairo 11321, Egypt

## Abstract

Cardiac arrest has been described in children with diabetic ketoacidosis (DKA). *Aim*. To evaluate QTc and QTd in type 1 diabetic children with DKA. *Methods*. Twelve-lead ECG was done to 30 type 1 diabetic children with DKA at presentation and recovery. Corrected QT interval and QT dispersion (QTd) were assessed. *Results*. QTc and QTd mean values were significantly decreased in patients after than before DKA recovery (*P* < 0.01). *Procedure*. Sixteen patients (53, 3%) had prolonged QTc during DKA (range 451–538 ms) that dropped to one patient after recovery, his QTc (453 ms) returned to normal 5 days after hospital discharge. Nineteen patients (63.3%) had prolonged QTd (>50 ms) that dropped to three after recovery. The fact that three patients had normal QTc but prolonged QTd increases the privilege of QTd over QTc as a better marker for cardiac risk in those patients. Anion gap was significantly associated with QTc and QTd prolongation (*P* < 0.0001). Patients had no electrolyte abnormalities or hypoglycemia to account for QTc or QTd prolongation. *Conclusion*. Prolonged QTc and QTd frequently occur in DKA positively correlated to ketosis. Cardiac monitoring for patients with DKA is mandatory.

## 1. Introduction 

Cardiac arrhythmias and arrest have been described in children with diabetic ketoacidosis and generally have been presumed to be caused by electrolyte abnormalities [1]. A previous study has reported a prolongation of QTc in children receiving ketogenic diets [2] and in other conditions associated with ketosis in absence of electrolyte [3]. The association between ketotic conditions and prolonged QTc and/or sudden death raises the suggestion of whether ketosis may directly affect cardiac repolarization and be a cause of arrhythmia and/or SCD in DKA patients. QT dispersion (QTd) is defined as the interlead variability in the duration of the QT interval in the 12-lead electrocardiogram [4]. It is a significant parameter that can be used to assess the homogeneity of cardiac repolarization and autonomic function. Prolonged QTd was previously suggested as the best predictor of cardiac death in patients with type 2 DM [5].

This study aimed to evaluate QTc and QTd in children with type 1DM during DKA.

## 2. Patients and Methods

This study included 30 children with type 1 DM who were presented with DKA 15 were newly diagnosed and 15 were following up in the diabetes clinic. They were 15 males and 15 females, their age ranged from 5 to 13 years with a mean age 9.59 ± 2.21 years (Table 1). DKA was defined according to ISPAD criteria [6]. Patients with congenital or rheumatic heart diseases, systolic left ventricular dysfunction, and unreliable identification of the end of the T wave in the ECG as well as patients taking medications that could affect QTc were excluded from the study. Patients were enrolled in the study after taken a written informed consent from parents or guardians. Patients were treated according to standard DKA protocol at presentation and ABGS, serum electrolytes (Na, K, Ca, Mg, Creatinine) were withdrawn, and the anion gap [Na −(Cl+ HCO3)] was assessed [6].

Twelve-lead ECG were done by a single pediatric cardiologist who was masked to patients' clinical and laboratory data. QTc was estimated according to Bazett's formula, where RR interval was measured in relation to the previous QRS complex $\text{QTC}=\sqrt{\text{QT}}/\text{R-R}\;\text{interval}$ (Bazett, 1920) [7]. The standard 12-lead ECG was recorded at 25 mm/s along with a lead II rhythm strip recorded at 50 mm/s; QT and RR intervals were measured. QT interval was measured from the onset of the QRS complex to the end of *T* wave. The end of QT interval was defined as the intersection of a tangent to the steepest downslope of the dominant repolarization wave with the isoelectric line. Lead II was used preferentially for QTc measurement [8]. Three separate measurements were obtained from each ECG, and the mean of these measurements was used as the value for the QTc. Prolonged QTc was defined as a QTc of at least 0.45 s (450 ms) [9]. QT-interval dispersion was defined as the difference between maximum and minimum non-rate-corrected QT-interval duration [10]. ECG was done first during DKA and another ECG after recovery from DKA. Follow-up ECGs was recorded at time of hospital discharge or at patient's first outpatient visit (median 22 days after initial ECG with a range from 5 to 30 days).

### 2.1. Statistical Analysis

Collected data were analyzed using SPSS program version 16 to obtain the quantitative variables as mean ± SD and range and qualitative variables as number and percentage. Unpaired (Student's) *t*-test and correlation coefficient test were performed. *P* values less than 0.05 were considered statistically significant and *P* values less than 0.01 were considered statistically highly significant.

## 3. Results

 QTc was found to be prolonged (more than 450 ms) in 16 patients: 9 patients with new onset diabetes and 7 patients with known diabetes during DKA (range 451–539 ms). The mean QTc values of patients during DKA were 450 ± 89 ms, which were significantly decreased after recovery from DKA (428 2 ± 5 ms, *P* < 0.001). Only one child had persistent prolonged QTc (453 ms) even after recovery from DKA which returned to normal 5 days after hospital discharge (Table 2). Nineteen patients (63.3%) had prolonged QTd (>50 ms): 10 were newly diagnosed patients with type 1 DM and 9 patients were known diabetics. Three children had persistent prolongation of QTd after recovery from DKA and were returned to normal within one week after hospital discharge. Three patients were found to have normal QTc (444 ms, 447 ms, and 445 ms, resp.) although they have increased QTd (51 ms, 52 ms, and 51, resp.). None of the children enrolled in the study experienced cardiac arrhythmias during DKA treatment. 

**Figure 1:**
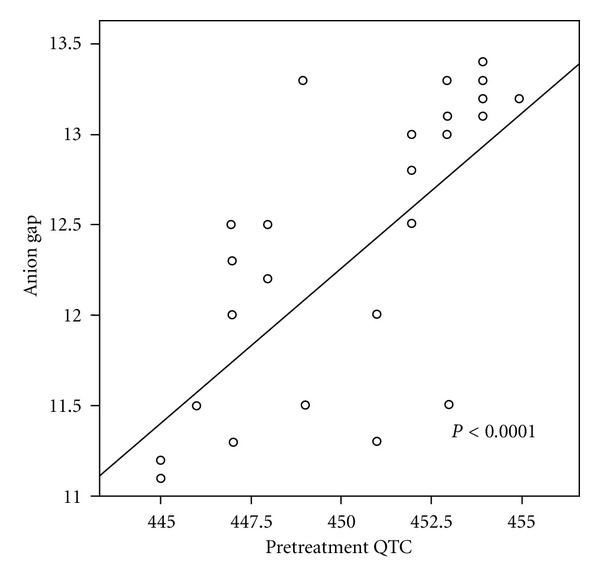
scatter diagram showing the positive relation between anion gap and pretreatment QTc.

During DKA, patients with prolonged QTc and those with prolonged QTd had a significant higher anion gap and lower PH at presentation compared to those with normal QTc and QTd values, while no significant difference was found between both groups of patients in other biochemical or clinical data (Tables 3 and 4). The initial anion gap was positively correlated with QTc values (*r* = 0.67, *P* < 0.0001) (Figure 1) and QTd value (*r* = 0.69, *P* < 0.001) during DKA. 

 None of children experienced hypoxia or hypoglycemia (blood glucose level <70 mg/dL), 5 patients had mild hypokalemia at the time of ECG recording (range from 3.1 to 3.3 mEq/L), while S. Mg^++^ and S. Ca were within reference range in all patients with no significant correlation between S. Ca, K, and Mg^++^ with QTc or QTd was found. 

## 4. Discussion

Arrhythmias and cardiac arrest have been reported as complications of DKA and generally have been presumed to be caused by electrolyte abnormalities [1]. In the present study, QTc and QTd prolongation were found in 16 and 19 patients, respectively, during DKA. After recovery, only one patient had persistent QTc prolongation and 3 had persistent QTd prolongation which returned to normal within one week following discharge. Since patients had no electrolyte disturbances (hypokalemia, hypomagnesemia and hypocalcemia) or hypoglycemia that could account for QTc prolongation, so the role of ketoacidosis in causing such prolongation and delayed cardiac repolarization was suggested. 

 Prolongation of QT interval is a serious condition that provides substrate for the development of potentially life-threatening arrhythmias torsade de pointes [11]. Previous studies supported the association of other ketotic conditions with QTc prolongation and deaths in patients receiving ketogenic diets [2, 9, 12].

 The fact that three patients who had normal QTc were found to have increased QTd increases the privilege of QTd as a better marker of cardiac risk than QTc in those patients. This result agreed with that reported by Rana et al. who found prolonged QTd to be the best predictor for cardiac death in patients with DM [13]. Moreover, Psallas et al. found that prolonged QTd interval may predict cardiac mortality in patients with diabetes and suggested that it may be a useful adjuvant index in the evaluation of cardiovascular risk in patients with type 2 diabetes and microalbuminuria [14].

Children with long-standing diabetes but without DKA reported to have prolonged QTc and a greater frequency of other abnormalities in cardiac autonomic function compared with age-matched control subjects. Those abnormalities were thought to be manifestations of diabetic neuropathy [15]. In the present study QTc prolongation could not be attributed to preexisting manifestations of diabetic neuropathy because QTc returned to normal after recovery from DKA in most of children. In addition, QTc prolongation was present in patients with new onset DM in addition to those with known diabetes. 

The anion gap was significantly greater at presentation in children with a prolonged QTc and QTd. In addition, a significant positive correlation was found between QTc and QTd values and initial anion gap in patients during DKA reflecting the role of ketosis in causing QTc and/or QTd prolongation, which agreed with Best et al. who described QTc prolongation in children receiving ketogenic diets [2].

 The only limitation of the present study is the use of anion gap as an indicator of ketosis without measuring serum ketonesconcentration. 

In conclusion, prolonged QTc occurs frequently during DKA and is correlated with ketosis. ECG and cardiac monitoring of children during DKA should be strictly followed.

## Figures and Tables

**Table 1:** Demographic and laboratory characteristics of the studied patients.

	Mean	±SD	Range	
Age in years	9.59	2.21	5.00	13.00
RBS (mg/dL)	433.00	51.69	350.00	600.00
pH	7.09	0.10	6.85	7.27
SK (mEq/L)	3.92	0.45	3.40	4.90
SCa (mg/dL)	9.27	0.26	8.80	9.70
SMg (mg/dL)	2.36	0.25	1.30	2.80
S. creatinine (mg/dL)	91	0.33	0.58	1.80
Anion gap	12.25	0.82	11.10	13.40
Pretreatment QTc (ms)	450	89	361	539
Posttreatment QTc (ms)	428.50	25	403.	453

Male-to-female ratio 1 : 1 (15/15).

**Table 2:** QTc and QTd values before and after treatment of DKA.

	Pretreatment	Posttreatment	*t* value	*P *
QTc (ms)	450 + 89	428.5 + 25	2.59	<0.01
QTd (ms)	48.77 + 6.36	41.97 + 8.1	7.70	<0.001

**Table 3:** Comparison between patients with prolonged and those with normal QTc before receiving treatment for DKA regarding age and laboratory data.

	Normal QTc (14)		Prolonged QTc (16)		*t *	*P *
	Mean	SD	Mean	SD		
Age in years	9.86	2.14	9.36	2.31	0.61	0.54
RBS (mg/dL)	401.43	34.85	405.42	33.82	0.67	0.61
PH	7.16	0.05	7.03	0.10	4.71	**0.0001**
S.K (mEq/L)	3.7	0.16	3.8	0.17	0.61	0.54
S.ca (mg/dL)	9.4	0.15	9.38	0.13	0.57	0.5
S.Mg (mg/dL)	2.53	0.18	2.52	0.2	0.52	0.34
S. createnine (mg/dL)	0.8	0.12	1.01	0.22	0.70	0.62
Anion gap	11.87	0.66	12.58	0.81	2.6	**0.01**

**Table 4:** Comparison between patients with prolonged and those with normal QTd as regards age and laboratory data.

	Normal QTd (11)		Prolonged QT (19)		*t *	*P *
	Mean	+SD	Mean	+SD		
Age in years	9.86	2.29	9.43	2.20	0.51	0.614
RBS (mg/dL)	401.27	35.54	407.89	34.44	1.21	0.54
PH	7.17	0.06	7.05	0.10	3.67	**0.001**
S.K (mEq/L)	3.69	0.18	3.44	0.12	1.19	0.54
S.ca (mg/dL)	9.44	0.11	9.47	0.16	1.25	0.30
S.Mg (mg/dL)	2.51	0.20	2.58	0.29	1.37	0.32
S. creatinine (mg/dL)	0.81	0.13	0.97	0.39	1.29	0.21
Anion gap	11.92	0.17	12.44	0.13	3.75	**0.001**
